# Demethylation effect of the antineoplaston AS2-1 on genes in colon cancer cells

**DOI:** 10.3892/or.2013.2839

**Published:** 2013-11-08

**Authors:** MASATAKA USHIJIMA, YUTAKA OGATA, HIDEAKI TSUDA, YOSHITO AKAGI, KEIKO MATONO, KAZUO SHIROUZU

**Affiliations:** 1 Department of Surgery, Kurume University School of Medicine, Kurume 830-0011, Japan; 2 Department of Surgery, Kurume University Medical Center, Kurume 839-0863, Japan; 3 Kurume Daiichi Social Insurance Hospital, Kurume 830-0013, Japan

**Keywords:** demethylation, epigenetic modification, antineoplaston AS2-1, colon cancer, silencing of tumor suppressor genes

## Abstract

Antineoplastons are naturally occurring peptides and amino acid derivatives found in human blood and urine. Antineoplastons have been shown to control neoplastic growth. In the present study, we investigated demethylation effect of the antineoplaston AS2-1 (a mixture of phenylacetylglutamine and phenylacetate in the ratio of 1:4) on various genes in colon cancer cells. An *Hpa*II-*Msp*I methylation microarray was used to investigate the methylation status of 51 genes at the promoter region in HCT116 and KM12SM human colon cancer cells before and after treatment of AS2-1. The expression of protein and mRNA of the demethylated genes by AS2-1 in HCT116 cells was evaluated. In 19 of the 34 methylated genes in HCT116 and in 7 of the 8 methylated genes in KM12SM, the methylation status was downregulated after treatment with 2 mg/ml of AS2-1 for 24 h. AS2-1 dramatically downregulated the methylation status of *p15* and *ESR1* in HCT116 cells and of *MTHFR* and *MUC2* in KM12SM cells. Both mRNA and protein expression of p15 increased in a dose- and time-dependent manner after treatment with AS2-1. The antineoplaston AS2-1 may normalize the hypermethylation status at the promoter region in various genes including tumor suppressor genes, resulting in activation of the transcription and translation in colon cancer.

## Introduction

Antineoplasons are naturally-occurring peptides and amino acid derivatives found in human blood and urine, first described by Burzynski in 1976 (1). Antineoplaston A10 (3-phenylacethyl-amino-2,6-piperidinedione) is the first chemically-identified antineoplaston and it is partially hydrolyzed in pancreatic juice to phenylacetylglutamine (PG) and phenylacethyl isoglutamine (isoPG) when administered perorally. PG and isoPG are further metabolized to phenylacetate (PN). The mixture of PG and PN in the ratio of 1:4 has been formulated as antineoplaston AS2-1. Antineoplastons have been found to control neoplastic growth. The animal experiment and phase I clinical toxicological studies (2) have demonstrated minimal adverse effects of these agents. Thus, it is postulated that combining these antineoplastons with intensive chemotherapy may increase antitumor efficacy without an increase of adverse effects in cancer patients. Clinical studies have described the antitumor efficacy of antineoplastons for various tumors including hepatocellular carcinoma, colon cancer and glioma (3–5).

Sodium phenylbutyrate (PB) and PN (a metabolite of PB) that is the active ingredient of antineoplaston AS2-1 induce cytostasis, differentiation and apoptosis by several cellular mechanisms, in glioma, neuroblastoma, leukemia cells and adenocarcimoma cells of the breast, colon and lung (6–9). PN activates the *p53* and *p21* genes through inhibition of methyltransferase and fernesylation of the RAS protein (10). PB activates tumor suppressor genes through inhibition of histone deacetylation (11,12). PG that is also the active ingredient of antineoplaston AS2-1 normalizes the pattern of genome-wide methylation, stabilizing the genes, decreasing expression of oncogenes such as *AKT-2* and *c-myc (MYCC)* and activating tumor suppressors proteins phosphatase and tensin homologue (*PTEN*) and integrase interactor 1 (*INI1*) and promotes apoptosis (13). Thus, one of the mechanisms underlying the antitumor effect of antineoplaston AS2-1 is considered to involve regulation of tumor suppressor gene expression through demethylation of their promoter sequences and modification (acetylation) of histones.

Epigenetic alterations of gene function are now well known to contribute to the tumorigenesis and cancer progression. Specifically, abnormal promoter region methylation which typically occurs at CpG islands in known or candidate tumor suppressor genes contributes to tightly heritable gene silencing and can thereby cause the loss of gene function. Silencing of genes is a complex process, which involves methylation of DNA, histone modification and chromatin remodeling. Two biochemical processes play a very important part in silencing of the genes: deacetylation of histones and methylation of DNA (14,15). Additional new mechanisms of methylation have been proposed explaining two different issues of DNA methylation in cancer progression: i) site-specific hypermethylation of promoter sequences; and ii) genome-wide hypo-methylation is inducing genomic instability, amplification of oncogenes and silencing of tumor suppressor genes through RNAi mechanism (16,17). Methylation of specific genes or methylation patterns of groups of genes were also found to be associated with responses to chemotherapeutics and prognosis.

The antitumor mechanisms on the epigenetic modification of antineoplastons have not yet been clarified. In the present study, we have investigated the epigenetic modification, in particular in demethylation effect of the antineoplaston AS2-1 and clarify sequential increase of transcription and translation to protein for targeting genes in colon cancer cell lines.

## Materials and methods

### Antineoplaston AS2-1

Antineoplaston AS2-1 injected formulation (20 g/250 ml) which is a mixture of PG and PN in the ratio of 1:4, was a kind gift from Dr S.R Burzynski (Burzynski Institute, Houston, TX, USA).

### Cell lines and culture

The HCT116 human colon cancer cell line (*p53* wild) was obtained from the ATCC (Rockville, MD, USA). The KM12SM human colon cancer cell line (*p53* mutant) was a kind gift from Dr Motowo Nakajima (SBI Arapromo K.K., Tokyo, Japan). Each cell line was cultured in RPMI-1640 medium (Invitrogen, Tokyo, Japan) supplemented with 10% fetal bovine serum (FBS) at 37°C/5% CO_2_ in 75-cm^2^ culture dishes. The cells were trypsinized once a week with trypsin/EDTA (0.25%/0.02) and the medium was changed twice a week.

### In vitro cell growth assay

The HCT116 and KM12SM cells were seeded at a density of 2.5×10^4^ into a 6-well dish containing 4 ml RPMI-1640 medium (Invitrogen, Carlsbad, CA, USA) supplement with 10% FBS and incubated overnight. The next day, AS2-1 was added to the subconfluent cultures at concentrations of 0.2, 0.5, 1, 2 and 5 mg/ml. The tumor cells were harvested before AS2-1 treatment as control, and after AS2-1 treatment at 24, 48 and 72 h. The number of viable tumor cells was determined by the trypan blue exclusion test.

### Evaluation of methylation profiles at promoter regions by HELP assay (HpaII tiny fragment enrichment by ligation-mediated PCR)

*Hpa*II-*Msp*I methylation microarray (Methyl-Scan DNA chip; Genomictree Inc, Daejeon, South Korea) was used to investigate the methylation status of 51 kinds of gene promoter region in HCT116 cells and KM12SM cells before and after treatment with 2 mg/ml of AS2-1 for 24 h. All chemical reagents used were purchased from Sigma-Aldrich (Haverhill, MA, USA) unless otherwise noted. *Hpa*II and *Msp*I restriction enzymes were obtained from New England Biolabs (Ipswich, MA, USA). Oligonucleotides were synthesized by Bioneer Inc. (Daejeon, South Korea).

To avoid incomplete digestion and reduce the background noise signals, 200 ng of genomic DNA was digested with excessive units of *Hpa*II and *Msp*I (80 units each) for 6 h at 37°C with enzyme buffers recommended by the suppliers. The digested samples were inactivated at 65°C for 20 min and then purified with GeneClean Turbo kit (Qbiogene, Irvine, CA, USA) according to the manufacturer’s instructions.

Multiplex PCR amplification was done with un-digested and *Hpa*II-, *Msp*I-digested DNA with primers sets to label 51 target promoter regions. The sequences of the gene-specific primer sets used are shown in Table I. During the amplification step, fluorescent dyes were incorporated into the amplicons; Cy3-dUTP in *Msp*I-digested targets, Cy5-dUTP in *Hpa*II-digested targets and undigested samples labeled with Cy5-dUTP by same multiplex PCR. The amplification was carried out according to the general guidelines: denaturating at 94°C for 5 min, followed by 30 cycles at 94°C for 30 sec, at 66°C for 45 sec, at 72°C for 45 sec and a final extension at 72°C for 5 min. To assess PCR adequacy and ensure scanning, the human interferon-2 gene without *Hpa*II site was used as a control.

After PCR amplification, all the amplicons were mixed and purified by using QIAquick PCR cleanup kit (Qiagen K.K., Tokyo, Japan). The hybridization mixture (100 μl) contained the amplified Cy5 and Cy3-labeled cDNA, 3.5X SSC, 5 μg of salmon sperm DNA and 0.2% sodium dodecyl sulfate. It was heated for 2 min at 95°C and immediately applied onto Methyl Scan DNA chip. The arrays were incubated at 65°C for 4 h in an eight-well platform hybridization chamber (Genomictree). To determine the methylation pattern, the hybridized microarray was imaged by an Axon 4000B scanner (Axon Instruments, Inc., Union City, CA, USA). The signal intensities were measured and analyzed by using GenePix Pro (version 4.0) software. If the signal intensity of *Hpa*II amplicon is 2-fold greater than that of *Msp*I amplicon, the target region was considered to be methylated, while <2-fold was considered to be unmethylated (minus). Moreover, the methylated status was categorized into 3 degrees, high priority (three plus), middle priority (two plus) and low priority (plus).

### Real-time RT-PCR

To clarify the reinforcement of a transcription activity of the methylate normalized gene by AS2-1, real-time RT-PCR (relative quantitative) for *p15* messenger RNA in HCT116 cells were evaluated. The HCT116 cells were seeded at a density of 2.5×10^4^/ml into a 6-well dish containing 4 ml RPMI-1640 medium (Invitrogen) supplement with 10% fetal bovine serum (FBS) and incubated overnight. The next day, AS2-1 was added to the subconfluent cultures at concentrations of 0.2, 1 and 2 mg/ml. The tumor cells were harvested before AS2-1 treatment as control, after AS2-1 treatment at 12, 24 and 48 h.

Isolated RNA was controlled for quality by 2% agarose gel separation and ethidium bromide staining. RNA was quantified by spectrophotometry. Complementary DNA (cDNA) was synthesized using 2 μg of total RNA. The 20 μl reverse transcription reaction consisted of 2 μl 10X RT buffer, 0.5 mM each dNTP, 1 μM Oligo-dT primers and 4 U Omniscript reverse transcriptase (Qiagen K.K). The reverse transcription reaction was incubated for 1 h at 37°C and then at 93°C for 15 min. Real-time PCR analysis was performed using the ABI Prism 7700 sequence detection system (Applied Biosystems, Foster City, CA, USA) as previously described (18). Briefly, reactions were performed in a 96-well optical reaction plated on cDNA equivalent to 50 ng of DNase-digested RNA in a volume of 25 μl, containing 12.5 μl of TaqMan Universal Master Mix and optimized concentrations of carboxy fluorescein (FAM)-labeled probe and forward and reverse primers following the manufacturer’s protocol. All primers and FAM-labeled probes for mouse *p15* (forward, 5′-TCTGCAGCTGGATCTGGTCC-3′ and reverse, 5′-TCCT GAAAGGTAGAGGGCCC-3′) and *GAPDH* (forward, 5′-CA TCTCCTCCCGTTCTGCC-3′ and reverse, 5′-GTGGTGC AGGATGCATTGC-3′) were obtained from Applied Biosystems. The mRNA expression of *p15* was normalized to the level of *GAPDH* mRNA. Data were processed by the RQ Manager 1.2 software.

### Western blot analysis

The cells were cultured as previously described. The cells were directly lysed in a sample buffer (0.5 M Tris-HCl, pH 6.8, 10% glycerol, 10% SDS, 6% mercaptoethanol, 0.05% bromophenol blue). Protein (10 μg) was separated on 4% SDS-PAGE gel at 120 V for 1.5 h. After electrophoresis, the proteins were transferred to a PVDF membrane (Bio-Rad Laboratories, Hercules, CA, USA) at 100 V for 60 min. The membrane was blocked in PBS containing 0.1% Tween-20 (FBS-Tween) with 5% skimmed milk at room temperature for 60 min, subsequently incubated with anti-p15 mouse monoclonal antibody (Abcam, Cambridge, UK) at 4°C overnight. After washing with PBS-Tween, the membrane was incubated with a horseradish peroxidase-conjugated goat anti-rabbit IgG or anti-mouse IgG secondary antibody (Santa Cruz Biotechnology, Dallas, TX, USA). The membrane was washed with PBS-Tween, and the signal was detected using an ECL detection kit (Amersham Pharmacia Biotech Inc., Piscataway, NJ, USA). A mouse monoclonal antibody against β-actin (Sigma-Aldrich, St. Louis, MO, USA) was used to control for protein loading. The amount of each protein was quantified as ratio to actin. Quantification of band densities was performed using the public domain NIH Image software.

## Results

### Cell growth inhibition by antineoplaston AS2-1

**Figure 1:**
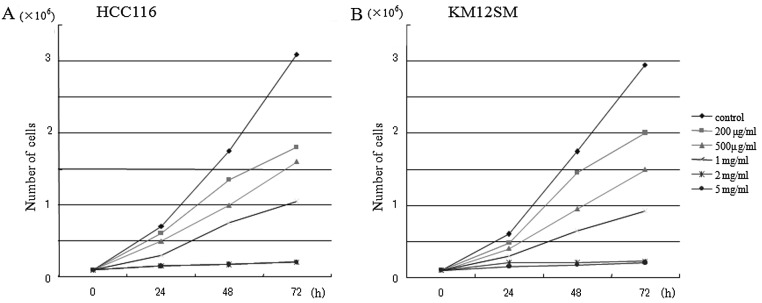
*In vitro* cell growth inhibition by antineoplaston AS2-1. Human colon carcinoma HCT116 and KM12SM cells were incubated as described in Materials and methods. Antineoplaston AS2-1 inhibited (A) HCT116 and (B) KM12SM cell growth in a time- and dose-dependent manner.

Antineoplaston AS2-1 inhibited the cell growth of KM12SM and HCT116 in a culture in a dosa- and time-dependent manner (Fig. 1). There was no difference in cell growth inhibition by AS2-1 between the wild *p53* HCT116 and the mutant *p53* KM12SM cell lines.

### Methylation status

Among the 51 genes, there were 34 methylated genes in promoter sequence in HCT116 cells. In 19 (56%) of the 34 methylated genes the methylation status was downregulated after treatment with 2 mg/ml of AS2-1 for 24 h, in particular, the methylation status of cyclin-dependent kinase inhibitor *p15 (INK4B)* and estrogen receptor 1 (*ESR1*) in HCT116 cells were dramatically downregulated from two plus to minus (Tables II and III). Among the 51 genes, there were 8 methylated genes in KM12SM cells. In 7 (88%) of the 8 methylated genes in KM12SM cells, the methylation status was downregulated after treatment with 2 mg/ml of AS2-1 for 24 h, in particular the methylation status of methylenetetrahydrofolate reductase (*MTHFR*) and mucin2 (*MUC2*) in KM12SM cells was dramatically downregulated from two plus to minus (Tables IV and V). There was no gene with methylation status upregulated in both HCT116 and KM12SM cells.

**Figure 2:**
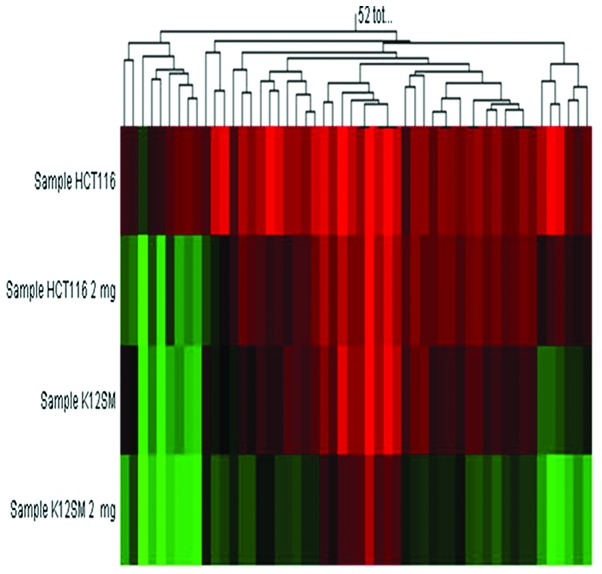
Multiple clustering analysis of methylation status. Red pixels represent methylation status: the intensities of red pixels depend on methylation status (priority). Greenish black pixels represent unmethylated status. Sample HCT116, before treatment; sample HCT116 2 mg, after treatment with 2 mg of antineoplaston AS2-1 for 24 h; sample KM12SM, before treatment; sample KM12SM 2 mg, after treatment with 2 mg of AS2-1 for 24 h

Of interest, in between the cell lines there was a difference in demethylation effect of AS2-1 in the 8 genes which were methylated in both HCT116 cells and KM12SM cells. Among the 8 genes, there was only one gene (*MUC2*) of which methylation status was downregulated after treatment with AS2-1 in HCT116 cells, whereas in 7 of the 8 methylated genes in KM12SM cells, the methylation status were downregulated by AS2-1 (Fig. 2).

### Transcription of p15 mRNA

**Figure 3:**
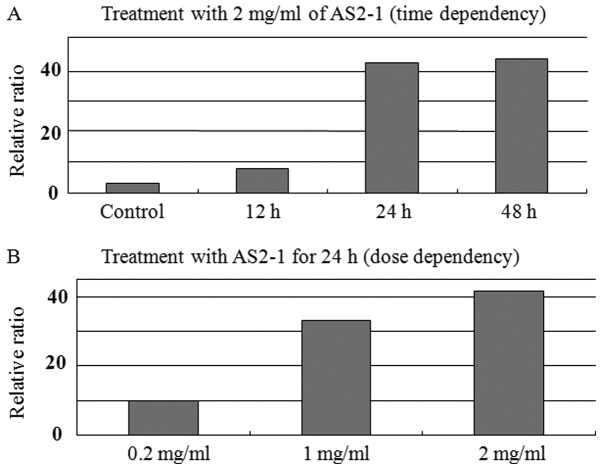
Real-time RT-PCR analysis for *p15* mRNA in HCT116 cells. (A) HCT116 cells were treated with 2 mg/ml of antineoplaston AS2-1 for 12, 24 and 48 h. (B) HCT116 cells were treated with 0.2, 1 and 2 mg/ml of AS2-1 for 24 h. AS2-1 upregulated the expression of *p15* mRNA in a time- and dose-dependent manner in HCT116 cells.

Real-time RT-PCR analysis revealed that AS2-1 upregulated the expression of *p15* mRNA in a time- and dose-dependent manner in HCT116 cells (Fig. 3).

### Translation of p15 protein

**Figure 4:**
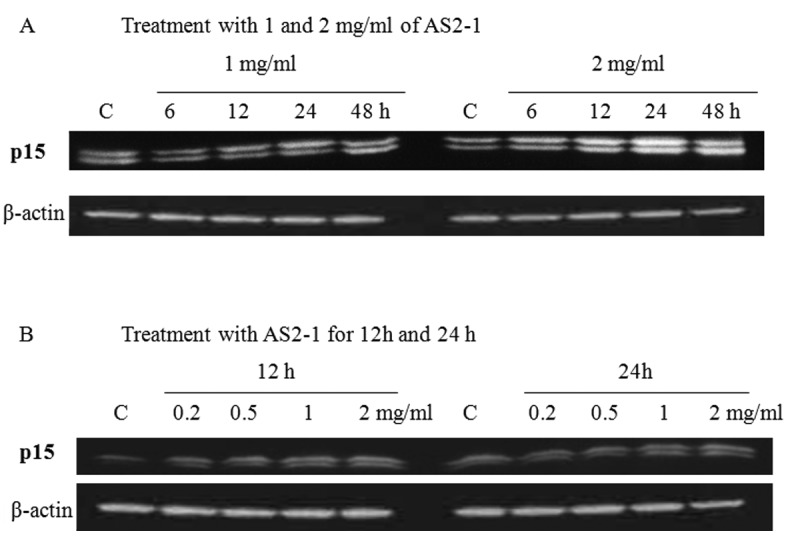
Western blot analysis for p15 protein in HCT116 cells. (A) HCT116 cells were treated with 1 mg/ml or 2 mg/ml of antineoplaston AS2-1 for up to 48 h. (B) HCT116 cells were treated with 0.2, 0.5, 1 and 2 mg/ml of AS2-1 for 12 or 24 h. AS2-1 upregulated the expression of p15 protein in a time- and dose-dependent manner in HCT116 cells.

Western blot analysis revealed that AS2-1 upregulated the expression of p15 protein in a time- and dose-dependent manner in HCT116 cells (Fig. 4).

## Discussion

Burzynski found compounds in the blood and urine of healthy adults that were absent in blood and urine of cancer patients, as first described in 1976 (1). He proposed the hypothesis that these peptide fractions could trigger normal differentiation and apoptosis in neoplastic cells and their deficiency in cancer patients contributed to disease progression. These peptide fractions were termed ‘antineoplastons’.

Re-expression of abnormally silenced suppressor genes is being investigated for potential clinical applications. Based on animal experiments and human observations, the drugs which can restore normal gene expression in aging should inhibit insulin-like growth factor 1 (IGF-1)/AKT and RAS pathways and provide proper anticancer defense through normal activity of tumor suppressors *p53* and *p21*(19). It has also been introduced that a group of amino acid derivatives and organic acids activate the tumor suppressors *p53*, *p21*, *PTEN* and *INI1* and decrease overexpression of *RAS* and *AKT-2* and *MYCC* oncogenes (20).

The current theory of the mechanisms of action of antineoplastons is that they function as ‘molecular switches’, turning on tumor suppressor genes and turning off oncogenes. It has been suggested that AS2-1 and PN activate tumor suppressor genes *p53* and *p21* through demethylation of their promoter sequences (21). In the present study, the methylation chip analyses revealed that AS2-1 downregulated methylation status at promoter sequences in various genes including well-known tumor suppressor genes and the candidates for tumor suppressor gene in both *p53* wild HCT116 and *p53* mutant KM12SM colon cancer cells. The results suggest that AS2-1 has a potent effect to normalize hypermethylation at promoter regions independently of *p53* mutation. Of interest, the demethylation effect was seen more completely in the KM12SM cells (8 methylated genes) than in the HCT116 cells (34 methylated genes). The difference in demethylation effect in the same genes between the two cell lines suggests that the demethylation effect of AS2-1 may depend on the cell type not on the genes. Cytidine analogs such as 5-azacytidine (azacitidine) and 5-azadeoxycytidine (decitabine) are the most commonly used demethylating agents. These compounds work by binding to DNA methyltransferases that catalyse the methylation reaction and titrate out DNA methyltransferases (22). If the demethylation effect of AS2-1 depends on activity of DNA methyltransferases, it is suspected that AS2-1 may demethylate at CpG islands of promoter region more completely in tumor cells with a low DNA methyltransferase activity.

One of the dramatically demethylated genes after treatment with AS2-1 in HCT116 cells was the tumor suppressor gene, cyclin-dependent kinase inhibitor *p15* (*INK4B*). Sequentially the expression of *p15* mRNA and p15 protein was upregulated by treatment with AS2-1. The results indicate that treatment with AS2-1 leads to re-expression of *p15* mRNA and p15 protein. This gene is localized to a region on chromosome 9p21 frequently deleted in human tumors. *p15* gene is an important mediator of cell cycle control, especially in pathways stimulated by TGF-β (23), raising the issue of whether inactivation of one or both of the genes and cyclin-dependent kinase inhibitor *p16 (INK4A)* with homozygous deletion involving the nearby *p15* is necessary, because both proteins are inhibitors of cyclin-dependent kinase 4 (INK4 family) (24,25) and are highly homologous throughout their coding sequence (26). It has been reported that the *p15* gene is commonly inactivated in association with promoter region hypermethylation involving multiple sites in a 5′-CpG island in glioma, leukemia and myelodysplastic symdromes. In other tumors, including lung, head and neck, breast, prostate and colon cancer, inactivation of *p15* occurs rarely and with concomitant inactivation of *p16*. It has been shown that aberrant methylation of *p15* is associated with transcriptional loss of this gene and treatment with the demethylating agent decitabine leads to reactivation of *p15*, inducing growth arrest and apoptosis in myeloid cell lines (27). AS2-1 may activate silenced tumor suppressor *p15* by demethylation and sequentially upregulates the expression of p15 protein in colon cancer HCT116 cells.

In conclusion, antineoplaston AS2-1 may normalize hypermethylation status at the promoter region in various tumor suppressor genes of which expression is silenced in colon cancer. Then, AS2-1 activates the gene transcription and protein translation, resulting in differentiation, cell cycle arrest and apoptosis in cancer cells.

## Figures and Tables

**Table I:** Primer information for *Hpa*II-*Msp*I-PCR assay.

Gene	Forward primer (5′-3′)	Reverse primer (5′-3′)	Amplicon size (bp)
ASIC2	ccaggaggcgaggagagaatg	cctcctctcctcctcctcca	178
Apaf-1	ccttggggcttggggtgtgt	cctcaagtcttcgcgggtcg	201
APC	caggcaacccagacgtccagag	ggaagttgatggcagttgacac	310
AR	ggacccgactcgcaaactgtt	gctggcgtggtgcgtccct	195
BRCA1	gcgtgagctcgctgagacttc	ccttcctgatcctcagcgctt	302
hCTR	ggatcagagttggaagagtccc	cctcccagcgccagcgact	382
CALCR	cctgtgtttacgcggcgcttt	gcagcagaattgatgagagcca	229
CDH13	ccatgcaaaacgagggagcgt	cgcacagaacgagcggagttc	258
CDKN2A (p16)	gcagcatggagccttcggct	ccaggaggaggtctgtgattac	292
CDKN2B (p15)	ccttggcccagctgaaaacg	gcactctctccttcctaggag	304
CFTR	cctccagcgttgccaactgg	cgtctgggctcaagctccta	443
COMT	cccattgctctgtgcagcct	ggctgggtgccttgtctaag	202
DAPK1	ccactcactccctagctgtgtt	cccagttgctcgaggcactgc	224
EDN1	ggtacacaggccatataggaac	ccgaatccctgggcatcagg	620
EBR	ggagggaacagcggtttccaa	cgtaacgggaggaatacagac	284
EPHA3	cctgtcccatgggcgacg	gctggtgcagagggcagtg	295
EPO	gcagcccccatgacccaca	gctgttatctgcatgtgtgcgt	175
ESR	cctggatccgtctttcgcgtt	gcagggtgcagaccgtgtcc	510
FHIT	ctccctccctctgcctttcat	ggcgatcccaccctgagacc	217
H19	gccatgtgcaaagtatgtgcag	cctggacagttccagcacac	228
Heparanase	gggagaggaagggatgaatact	ggtcacgttcacttacgaaatca	273
hMLH1	cgggcagtacctctctcagcaac	ggcttgtgtgcctctgctgagg	528
HTR1B	gggagcttccttggccagga	ctctgccctccctctcttttc	434
IL-8	ggaagtgtgatgactcaggttt	ggctcttgtcctagaagcttgt	195
JunB	ggaaacgacgccaggaaagct	gcagcgagcggcgagctct	174
LAMA5	ggcacaggctgactcatgtgt	gcttgcaggctgaccgcct	192
LDHB	gggagtgtgcacacttgagc	ctaccaggagagagaaggctc	237
BLT1	gtgagcgccatcgtgcttgc	caccactttcagctgagggg	332
LRP2	cgtgtgcacgtgtgagtgtg	cctctgctagcgaacgctcc	485
MAGE1	gcagagagagagtcttggctttc	cttgactgccgaccagtcctg	501
MDR3	cctaggagtactcacttcagg	cctctgcttctttgagcttgg	230
MGMT	ccgtttgcgacttggtgagt	ggaaaggctgggcaacacctg	199
MTHFR	gctgcctgccccctgatgc	ccccaggcaccaccactcc	346
MUC2	ggttggtcctcccagcgtaa	cctggcaggagggtaggag	239
PGR	ggtagggaggggctttggg	ccagcgagcggcaagtggg	579
PIK3CG	ccctctggggcattcattacta	ggaagcactacagcccttcag	139
PLS3 (TLS3)	cacccagttgatgtgacaggc	gctccaactgaaatttctccga	393
PTGS2	cccatccaaggcgatcagtc	ggtaggctttgctgtctgagg	471
RAR-b	gtgacagaagtagtaggaagtga	ccaggcttgctcggccaatc	338
RB1	ggatagggatgaggcccaca	cgtcccctgagaaaa accgg	341
S100A2	ccacagttctctcattccagc	ctcaggattctttttgcagcaac	578
SHP1	gctctgcttctcttcccttgc	gggactaagcctcagatgcag	193
SKT11 (LKB1)	cgaggacgaagttgaccctg	ggacccagggtcctggagt	324
NIS	cccagtccagggctgaaagg	ctccctgggttaggaatctatg	468
HLTF	ggagacggcgtcgacgtct	cgctgagtgggatgacaagag	181
SRBC	gcagtggagacctgaaacagg	ctggctgcactacggtcagg	286
TFF1	ctcagatccctcagccaagata	cgagtcagggatgagaggcc	229
TP73	gcctttggcgccaaagacagc	cgaaaccgcttagtaaccaactc	308
TUSC3 (N33)	ccttcatcatccaagaaggcatt	cgaggacgcagagtaggaga	295
VHL	cgagttggcctagcctcgc	cgtcttcttcagggccgtac	311
WT1	gctgctgagtgaatggagcg	gggtgaatgagtaggtgggag	293
IFN (control)	gcagctgcagcagttccaga	tgctcatgagttttccctggt	221

**Table II:** Methylation status in HCT116 cells before and after treatment with AS2-1.

Apaf-1	APC	AR	ASIC2	BLT1	BRCA1	CALCA
(+) ⇒ (−)	(+)⇒ (−)	(+)⇒ (−)	(−) ⇒ (−)	(+++)⇒ (+++)	(−) ⇒ (−)	(−) ⇒ (−)
CDH13	CFTR	COMT	DAPK	EBR	EDN1	EphA3
(+)⇒ (+)	(−) ⇒ (−)	(−) ⇒ (−)	(+)⇒ (+)	(+)⇒ (−)	(−) ⇒ (−)	(−) ⇒ (−)
EPO	ESR1	FHIT	hCTR	Heparanase	HLTF	hMLH1
(+)⇒ (−)	(++)⇒ (−)	(+)⇒ (−)	(+)⇒ (+)	(+)⇒ (+)	(−) ⇒ (−)	(+)⇒ (−)
HTR1B	H19	IL-8	JunB	Laminin5	LDHB	LKB
(+)⇒ (−)	(+)⇒ (+)	(−) ⇒ (−)	(+)⇒ (+)	(−) ⇒ (−)	(+)⇒ (−)	(+)⇒ (−)
LRP2	MAGE	MDR3	MGMT	MTHFR	MUC2	NIS
(+)⇒ (−)	(+)⇒ (−)	(−) ⇒ (−)	(−) ⇒ (−)	(++)⇒ (++)	(++)⇒ (+)	(+)⇒ (+)
N33	PGR	PIK3CG	PTGS2	P15	P16	P73
(+)⇒ (−)	(−) ⇒ (−)	(−) ⇒ (−)	(−) ⇒ (−)	(++)⇒ (−)	(−) ⇒ (−)	(+)⇒ (+)
RAR-β	RB1	SHP1	SRBC	S100A2	TFF1	TLS3
(+)⇒ (−)	(+)⇒ (+)	(+)⇒ (−)	(+)⇒ (−)	(+)⇒ (−)	(−) ⇒ (−)	(+)⇒ (+)
VHL	WT1					
(+)⇒ (+)	(+)⇒ (+)					

(Before treatment) ⇒ (After treatment: antineoplaston AS2-1 2 mg/ml, 24 h). If the signal intensity of *Hpa*II amplicon is 2-fold greater than that of *Msp*I amplicon, the target region was considered to be methylated, while <2-fold was considered to be unmethylated (minus). Moreover, the methylated status was categorized into 3 degrees, high priority (three plus), middle priority (two plus) and low priority (plus).

**Table III:** Summary of methylation status in HCT116 cells.

Before treatment		After treatment		
		
Status	n	Status	n	(%)
(−)	17	(−)	17	100
(+)	29	(+)	13	45
		(−)	16	55
(++)	4	(++)	1	25
		(+)	1	25
		(−)	2	50
(+++)	1	(+++)	1	100

**Table IV:** Methylation status in KM12SM cells before and after treatment with AS2-1.

Apaf-1	APC	AR	ASIC2	BLT1	BRCA1	CALCA
(−) ⇒ (−)	(−) ⇒ (−)	(−) ⇒ (−)	(−) ⇒ (−)	(+++)⇒ (+++)	(−) ⇒ (−)	(−) ⇒ (−)
CDH13	CFTR	COMT	DAPK	EBR	EDN1	EphA3
(+)⇒ (−)	(−) ⇒ (−)	(−) ⇒ (−)	(−) ⇒ (−)	(−) ⇒ (−)	(−) ⇒ (−)	(−) ⇒ (−)
EPO	ESR1	FHIT	hCTR	Heparanase	HLTF	hMLH1
(−) ⇒ (−)	(−) ⇒ (−)	(−) ⇒ (−)	(+)⇒ (−)	(−) ⇒ (−)	(−) ⇒ (−)	(−) ⇒ (−)
HTR1B	H19	IL-8	JunB	Laminin5	LDHB	LKB
(−) ⇒ (−)	(+)⇒ (−)	(−) ⇒ (−)	(−) ⇒ (−)	(−) ⇒ (−)	(−) ⇒ (−)	(−) ⇒ (−)
LRP2	MAGE	MDR3	MGMT	MTHFR	MUC2	NIS
(−) ⇒ (−)	(−) ⇒ (−)	(−) ⇒ (−)	(−) ⇒ (−)	(++)⇒ (−)	(++)⇒ (−)	(−) ⇒ (−)
N33	PGR	PIK3CG	PTGS2	P15	P16	P73
(−) ⇒ (−)	(−) ⇒ (−)	(−) ⇒ (−)	(−) ⇒ (−)	(−) ⇒ (−)	(−) ⇒ (−)	(−) ⇒ (−)
RAR-β	RB1	SHP1	SRBC	S100A2	TFF1	TLS3
(−) ⇒ (−)	(+)⇒ (−)	(−) ⇒ (−)	(−) ⇒ (−)	(−) ⇒ (−)	(−) ⇒ (−)	(−) ⇒ (−)
VHL	WT1					
(−) ⇒ (−)	(+)⇒ (−)					

(Before treatment) ⇒ (After treatment: antineoplaston AS2-1 2 mg/ml, 24 h). If the signal intensity of *Hpa*II amplicon is 2-fold greater than that of *Msp*I amplicon, the target region was considered to be methylated, while <2-fold was considered to be unmethylated (minus). Moreover, the methylated status was categorized into 3 degrees, high priority (three plus), middle priority (two plus) and low priority (plus).

**Table V:** Summary of methylation status in KM12SM cells.

Before treatment		After treatment		
		
Status	n	Status	n	(%)
(−)	43	(−)	43	100
(+)	5	(+)	0	
		(−)	5	100
(++)	2	(++)	0	
		(+)	0	
		(−)	2	100
(+++)	1	(+++)	1	100
